# The Effect of an 8 Week Prescribed Exercise and Low-Carbohydrate Diet on Cardiorespiratory Fitness, Body Composition and Cardiometabolic Risk Factors in Obese Individuals: A Randomised Controlled Trial

**DOI:** 10.3390/nu12020482

**Published:** 2020-02-14

**Authors:** Maria Perissiou, Erika Borkoles, Kent Kobayashi, Remco Polman

**Affiliations:** 1 School of Sport, Health and Exercise Science, University of Portsmouth, Portsmouth PO1 2UP, UK; 2 School of Medicine (Public Health), Griffith University, Gold Coast 4222, Australia; erika.borkoles@qut.edu.au; 3 School of Exercise & Nutrition Sciences, Queensland University of Technology, Brisbane 4059, Australia; kr.kobayashi@qut.edu.au (K.K.); remco.polman@qut.edu.au (R.P.)

**Keywords:** low carbohydrates, ketosis, prescribed exercise, cardiometabolic risk, β-hydroxybutyrate

## Abstract

Background: Low-carbohydrate (LC) diets are an effective method for treating obesity and reducing cardiometabolic risk. However, exposure to LC diets is associated with reductions in muscle mass and increased osteoporosis risk in obese individuals. The combination of exercise with a LC diet appears to attenuate muscle mass loss induced by LC diets alone, and to further improve cardiometabolic profile. However, evidence to date in obese individuals is limited. We assessed the effect of LC diet in combination with supervised exercise on cardiorespiratory fitness, body composition and cardiometabolic risk factors in obese individuals. *Methods:* Male and female participants in the experimental (EX-LC; structured supervised exercise program + low-carbohydrate meals; n = 33; 35.3 years) and control (EX-CO; structured supervised exercise program + standard dietary advice; n = 31; 34.2 years) conditions underwent measurements of cardiorespiratory fitness (*V*O_2_peak), body fat, lean muscle mass (LMM), and cardiometabolic biomarkers before and after an 8 week intervention. *Results*: Participants in the EX-LC condition demonstrated greater improvements in *V*O_2_peak (*p* = 0.002) and fat mass index (FMI, *p* = 0.001) compared to the EX-CO condition. Achieving a ketogenic state (β-hydroxybutyrate, βHB ≥0.3 mmol/L) was associated with greater reductions in total body fat (*p* = 0.011), visceral adipose tissue (*p* = 0.025), FMI (*p* = 0.002) and C-reactive protein (CRP, *p* = 0.041) but also with greater reductions in LMM (*p* = 0.042). *Conclusion*: Short-term LC diet combined with prescribed exercise enhanced cardiorespiratory fitness and the cardiometabolic profile of obese individuals but was also associated with greater muscle mass loss compared to similar exercise training and standard dietary advice. The long-term effects of the LC diet should be further explored in future studies.

## 1. Introduction

Obesity and related cardiometabolic risk factors associated with cardiovascular disease have dramatically increased globally over the recent years [1]. Obesity is a major public health challenge in Australia and its prevalence for 2019 is estimated to be 67% (32% obese, 34% overweight) [2]. In Queensland, two in three adults (66%) were overweight or obese in 2017–2018 [2], signifying a persistent public health problem. To date, no country has successfully reversed this trend [3]. Population-based preventive measures have been mostly unsuccessful [4]. Hence, effective treatment programs for those who are already overweight or clinically obese are needed to reduce weight and improve their cardiometabolic profile.

Ketosis is an alternative energy state when glucose levels are low [5]. Very low-carbohydrate diets, or ketogenic diets are becoming widely recognized as an effective method for treating obesity and managing weight [6]. Evidence suggests that low-carbohydrate meals lead to greater reductions in body fat and cardiometabolic risk factors, such as fasting glucose levels triglycerides and blood pressure, compared to standard low-fat meals [7,8,9]. This evidence suggests that once tissues become fat adapted utilising ketone bodies as a primary fuel source, profound cardiometabolic benefits emerge.

Despite the cardiometabolic benefits and the greater reduction in body fat, exposure to low-carbohydrate diets has been associated with various adverse outcomes including decreased muscle mass [10] and reduced bone mass [11]. Carbohydrate restriction leads to decreases in blood glucose. It is possible that increased gluconeogenic activity could promote the breakdown of muscle tissue and a reduction in muscle glycogen stores [12]. Reduced muscle mass is an established risk factor for osteoporosis [13], and has been associated with functional impairment in obese individuals [14]. Interestingly, the combination of exercise with a low-carbohydrate diet appears to attenuate muscle mass loss induced by ketosis [15]. However, evidence to date in obese and overweight individuals is limited.

Exercise training has been shown to significantly reduce cardiometabolic risk factors [16]. In particular the combination of aerobic and resistance training has been associated with increased mitochondrial biogenesis [17], improved vascular function, lipidaemic profile and reduced inflammation [18]—all of which are factors known to reduce cardiometabolic disease risk [19]. However, to date, no study has assessed the combined effect of a low-carbohydrate diet and prescribed exercise on cardiometabolic factors in overweight and obese individuals.

Therefore, the aim of the current study was to assess how an 8 week exercise intervention, consisting of aerobic and resistance exercise, in combination with a low-carbohydrate diet would improve body composition and cardiometabolic risk factors compared to the same exercise intervention combined with the standard Australian dietary guidelines in obese individuals.

## 2. Methods

### 2.1. Participants

**Figure 1:**
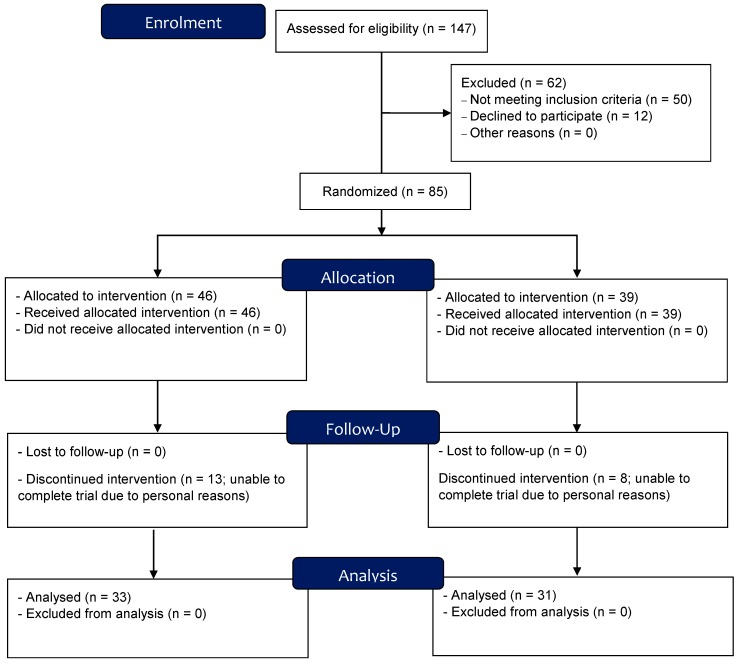
Consort flow diagram of the ‘Healthy Eating and Living Study’ (HEALS) trial.

A total of 64 obese men and women (BMI, 30.3 ± 3 kg.m^−2^; aged, 35.3 ± 9) were recruited from the local community to the ‘Healthy Eating and Living Study’ (HEALS) before being allocated to two conditions by randomised letters: Experimental (structured exercise programme + low-carbohydrate meals; EX-LC) or control (structured exercise programme + standard dietary advice; EX-CO) condition (Figure 1). Block randomization stratification by gender was undertaken, so that blocks of 12 participants were recruited at a time, randomized into one experimental condition of six participants and one control condition of six participants. Assessors prepared the envelopes with six paper codes (three experimental and three control group) which were added to opaque not concealed envelopes. There were two envelopes: one for females and one for males. Participants were asked to pick one paper from their respective envelope pack and the picked paper would either assign the participant to the experimental or control group. Participants were included if they were aged between 18 and 50 years at enrolment; had a body mass index (BMI) in the range of 30–35 Kg m^−2^ and no history of bariatric surgery. Participants were excluded if they had been diagnosed of any metabolic or cardiovascular disorder; thyroid disease, autoimmune or neurological disease; had uncontrolled hypertension; were a smoker; were pregnant or breast-feeding or were deemed unfit to exercise by a physician. Participants that were taking drug medication for thyroid, hyperlipidaemia, hypoglycemia, or were taking weigh loss supplements within 3 months before the start of the trial were also excluded. Participants were informed of the methods and study design verbally and in writing before providing written informed consent. The study conformed to the Declaration of Helsinki and was approved by a National Health and Medical Research Council (NHMRC) accredited ethics committee (Bellbery Limited HREC). In addition, the study was registered with the Australian New Zealand Clinical Trials Registry (No.: U1111-1206-4798).

### 2.2. Study Design Overview

Eligible participants attended the exercise physiology laboratory at the Institute of Health and Biomedical Innovation at Queensland University of Technology at baseline (BL) and 8 weeks after the experimental intervention (POST) each following an overnight fast. Participants were also asked to refrain from alcohol and exercise for 24 h, and caffeine for 12 h, before each visit, as these parameters have been shown to affect primary outcomes of the study [20,21]. The laboratory visits included an incremental maximal cycling test for the determination of cardiorespiratory fitness (*V*O_2peak_) and a body composition assessment via dual-energy X-ray absorptiometry (DXA), and a venous blood sample was drawn from an antecubital vein in order to assess lipid profile and β-hydroxybutyrate levels. Data collection of all the experimental measurements was performed on the same day for each participant. All participants followed an 8 week (4 sessions per week/45 min per session) prescribed exercise program. The exercise sessions involved a combination of aerobic and resistance exercise that included bouts of moderate [60%–70% HR peak/perceived exertion (RPE) 11–13 on Borg scale] [22] and high intensity exercise (85%–95% HR peak/RPE 15–17 on Borg scale). During the first 2 weeks of the intervention all sessions were supervised by an exercise physiologist, whilst only one session per week was supervised during weeks 3 to 8. Accredited clinical-exercise physiologists were trained on the prescribed experimental intervention and provided the intervention at the Health Clinics at QUT. The principal investigator of the current trial provided overall supervision of the experimental intervention. Participants were asked to keep a weekly exercise audio diary.

### 2.3. Dietary Intervention

Participants in the control condition were provided with written information on Australian Dietary guidelines as directed by the NHMRC Australia (NHMRC, 2013). Participants in the experimental condition were provided with 3 pre-prepared meals and 2 interim mid-meal snacks daily during the duration of the trial. A handbook containing recipe examples and food lists based on each experimental condition was also provided to all participants. Neither diet included a specific calorie or energy goal. For implementing controlled feeding protocols for the participants in the EX-LC group, we established a partnership with Thrive Collective Pty Ltd. (Sydney, NSW, Australia), a food service contractor, which provided the pre-prepared meals for the EX-LC group. The low-carbohydrate group pre-prepared meals did not exceed in total 50 g of CHO per day. All foods and quantities consumed were recorded daily for both groups, adherence to the diet was recorded daily via a 24 h food log. Alcoholic beverages were restricted for the intervention period in order to avoid any hyperglycemic incidents in the EX-LC group [23]. Dietary supplements were not permitted for the 3 months before and during the intervention period. Participants were asked to keep a weekly diet audio diary.

### 2.4. Maximal Incremental Cycling Test

This test was performed on an electromagnetically braked cycle ergometer (Lode Corival, Groningen, The Netherlands). Following a 3 min warm up (0 W), the test began at 60 W and then increased by 20 W each minute until volitional cessation. Participants self-selected a pedal cadence (>60 rpm) and maintained this throughout the test. Expired gases were collected continuously, and data were averaged every 15 s (Parvo Medics, Salt Lake City, UT, USA) for the determination of oxygen consumption (*V*O_2_; mL·kg^−1^·min^−1^). Peak cardiorespiratory fitness was determined as the highest 15 s average of *V*O_2_ over the last 60 s of maximal exercise (*V*O_2_peak). Heart rate was measured continuously using a polar heart rate monitor (Polar H10, Kempele, Finland) and recorded, along with perceived exertion (RPE) using the 6–20 Borg scale [22], during the final 10 s of each stage.

### 2.5. Body Composition Assessment

Body height was assessed by a standard stadiometer. Body weight was assessed by a digital scale (OMRON, VIVA, Hoffman Estates, IL, USA) before and after intervention. Body fat [total body and regional (VAT; visceral adipose tissue) fat distributions (%)], body mass index (BMI), fat mass index (FMI), lean muscle mass [LMM (cm^3^)] and total body mineral density [TBMD (g/cm²)] were evaluated following a 12 h overnight fast through DXA (DXA; Hologic QDR 4500 version 12.6) before and after intervention.

### 2.6. Blood Analysis

Samples were collected in K2 EDTA VacutainerTM tubes using standard aseptic techniques. Plasma was separated by centrifugation (1500× *g* for 15 min at 22 °C) and stored in 1.5 mL aliquots at −80 °C until further analysis. Fasting blood was analysed before and after intervention for Glucose (BGL), total cholesterol (TC), high density lipoprotein (HDL-C), Triglycerides (TG), C-reactive protein (CRP), low density lipoprotein (LDL-C), Adiponectin and ketone bodies (β-hydroxybutyrate). TC, HDL-C, TG, and CRP results were obtained by spectrophotometric assays on a Cobas Integra 400 (Roche, Rotkreuz ZG, Switzerland); LDL-C was then calculated by the Friedewald formula using the measured TG, TC, and HDL-C results. Adiponectin was measured by enzyme-linked immunosorbent assay (ELISA) (Millipore, Billerica, MA, USA) and ketone bodies (β-hydroxybutyrate, βHB) were obtained on a Beckman Coulter AU400 analyser (Beckman Coulter, Brea, CA, USA) utilising a RANBUT Test Kit (Randox, Crumlin, UK).

### 2.7. Resting Blood Pressure

Brachial systolic and diastolic blood pressure was measured using a manual analogue sphygmomanometer (Omron, Exactus Aneroid Sphygmomanometer, Melbourne, Australia) before and after intervention after ten minutes of seated rest.

## 3. Statistical Analysis

Based on the reported effect size (ES = 7.9 ± 1.6) on a previous metanalysis that assessed percent change of body fat in diet and exercise conditions [24], the current study adopted a conservative effect size of d = 0.80, with alpha at 0.05, and power at 0.90 which resulted in a sample size of 30 participants for each condition. The data were normally distributed. Our assessment of kurtosis and skewness of the participants’ age and body composition revealed values between −2 and 2 and therefore our data distribution is considered normal [25]. A single-factor linear mixed model was used to compare anthropometric characteristics, blood biomarkers and cardiorespiratory fitness between the experimental and control conditions. A two-factor (condition*time) linear mixed model was used to detect differences in body composition, blood biomarkers and cardiorespiratory fitness across time (before and after trial) and between conditions. Post-trial data were also analysed as changes from baseline (delta) to account for individual baseline variance. Statistically significant interactions were further investigated with multiple comparisons using Fisher’s least significant difference approach [26]. Our cohort was further stratified based on the β-hydroxybutyrate (βHB) ketone levels (βHB of 0.3 mmol/L≥ achieved ketosis; BHB of 0.3≤ not in ketosis) [5] and a multiple regression analysis was performed to investigate the mechanisms influencing the observed post-intervention change in the examined indices. Analyses were conducted using the Statistical Package for Social Sciences (Version 22; IBM SPSS Inc., Chicago, IL, USA) and statistical significance was set at *p* ≤ 0.05. Data are presented in the text and Tables as the mean and standard deviation (SD) unless otherwise stated.

## 4. Results

Participants’ characteristics of the complete cohort and comparisons between the EX-CO and EX-LC conditions are presented in Table 1. Cardiorespiratory fitness, body composition and other cardiometabolic indices are presented at Table 2 at baseline and following EX-CO and EX-LC conditions. Findings are summarised below.

### 4.1. Cardiorespiratory Fitness

Cardiorespiratory fitness measured as *V*O_2peak_ was not different between conditions at baseline (*p* = 0.46). Following both conditions, cardiorespiratory fitness significantly increased compared to baseline by 2.4 mL·kg^−1^·min^−1^ (95% CI 1.4 to 3.4, *p* = 0.001, Table 2). A condition effect revealed a greater increase in delta *V*O_2_peak after the EX-LC condition compared to after the EX-CO condition [mean diff of 3.4 mL·kg^−1^·min^−1^ (95% CI 1.3 to 5.5, *p* = 0.002)].

### 4.2. Body Composition

Body composition indices were similar at baseline between both conditions (*p* > 0.05). Table 2 demonstrates significant changes across time in the examined body composition indices following both of the conditions. Participants in EX-LC condition achieved a greater reduction in delta FMI [mean diff −0.7 kg.m^−2^ (95% CI −1.3 to 0.1, *p* = 0.013)] and in delta LMM [mean diff −1048 cm^3^ (−172 to 1923, *p* = 0.020)] compared to participants in EX-CO condition (Table 2).

### 4.3. Cardiometabolic Indices

Cardiometabolic indices were similar at baseline between the examined conditions (*p* > 0.05). Overall, total blood pressure, TC, TG, BGL and CRP were decreased following both conditions compared to baseline whereas negligible changes were observed in HDL, LDL and adiponectin (Table 2). Participants in EX-LC condition achieved a greater reduction in fasting delta BGL [mean diff −0.23 mmol/L (95% CI −0.02 to 0.42, *p* = 0.027)], delta TG [mean diff −0.41 mmol/L (95% CI −0.71 to 0.38, *p* = 0.045)] and delta CRP [mean diff −0.7 mmol/L (95% CI −1.4 to 0.3, *p* = 0.039)] compared to participants in the EX-CO condition.

### 4.4. Mixed Linear Regression Analysis

A mixed linear model regression analysis was used to further investigate the contribution of ketosis towards changes in cardiorespiratory fitness, body composition and cardiometabolic factors (Table 3). The increase in cardiorespiratory fitness (*V*O_2_peak) was associated ketogenic alterations (*p* = 0.026). Reaching a ketogenic state was also associated with a significant decrease in total body fat % (*p* = 0.011), VAT (*p* = 0.025), FMI (*p* = 0.002) and LMM (*p* = 0.042). Ketosis was also associated with reductions in CRP (*p* = 0.041) and BGL (*p* = 0.042) but not with changes in any of the other cardiometabolic indices (Table 3).

## 5. Discussion

The current study demonstrates that exercise training in combination with a low-carbohydrate diet, resulted in greater reductions in cardiometabolic indices, such as percent of body fat, triglycerides, blood glucose and inflammation and a greater increase in cardiorespiratory fitness compared to similar exercise training while on standard dietary advice. Although low-carbohydrate diet induced a significant reduction in lean muscle mass of ≈1.5%, no corresponding changes were observed in bone mineral density.

### 5.1. Cardiorespiratory Fitness

Despite following a similar exercise protocol, the EX-LC condition demonstrated a greater post-trial improvement in cardiorespiratory fitness normalised to body weight (*V*O_2_peak; mL.kg^−1^.min^−1^), compared to EX-CO condition. Previous studies in athletes reported similar results, showing that low-carbohydrate diet does not compromise aerobic capacity [27,28]. This greater relative improvement in cardiorespiratory fitness observed in the current study should be interpreted with caution, as a greater decrease in body mass was observed for the EX-LC compared to the EX-CO group. However, a study of Brinkworth et al. (2009) that assessed the effect of low-carbohydrate diet on aerobic capacity in obese individuals, reported no changes in cardiorespiratory fitness, despite the greater decrease in body mass observed in the low-carbohydrate group compared to the control group [29]. Hence, observed changes in body mass may not necessarily correspond with changes in cardiorespiratory fitness.

The current study demonstrated that the observed increase in *V*O_2_peak was associated with a plasma concentration of βHB ≥ 0.3 mmol/L, which suggests nutritional ketosis [30]. It has been demonstrated that a low-carbohydrate diet applied for at least four weeks results in a metabolic substrate shift towards a greater reliance on fat versus carbohydrate oxidation [31,32]. Increased fat oxidation has been shown to increase oxygen transport to the working muscles [33] and to reduce lactate concentration during maximal effort [34]—both of which are factors that affect cardiorespiratory fitness directly [35,36]. Hence, the greater cardiorespiratory fitness increase observed in the EX-LC group may be due to low carbohydrate availability. However, the current study did not measure metabolic rate directly and, hence, further research is needed regarding the effect of combined low-carbohydrate diet and exercise on cardiorespiratory fitness.

### 5.2. Body Composition

In the current study, all the examined body fat indices (total body fat, visceral adipose tissue and fat mass index) decreased significantly in both groups, which is in agreement with previous studies that have explored the effect of combined exercise in body composition in obese individuals [37,38]. Interestingly, and in agreement with a recent meta-analysis that assessed the impact of low-carbohydrate diet on body composition [39], increased levels of βHB were associated with greater post-trial reductions in the examined body fat indices. This could be attributed to the greater post-trial reduction observed in the blood glucose levels of the EX-LC group. Due to low levels of blood glucose induced by ketosis, stimulus for insulin secretion is also low, which sharply reduces the stimulus for fat storage [40].

A clinically significant finding of the current study is that increased levels of βHB were associated with a greater decrease in visceral adipose tissue. Visceral adiposity is an independent component of metabolic syndrome [41] and cardiovascular disease [42,43]. Preferential deposition of fat in the abdominal region predisposes an individual to atherogenesis and poor metabolic profile, as visceral adipose tissue releases free fatty acids into the hepatic circulation, thus stimulating an increase in lipoproteins [44] and plasma glucose [45]. Hence, the greater reduction observed in triglycerides and blood glucose in the EX-LC group in the current study, may be due to the greater reduction in visceral adipose tissue.

Consistent with the majority of the studies [29,45,46], the EX-LC group demonstrated a significantly greater reduction in lean muscle mass (LMM) compared to the EX-CO group. Similar to our results, Noakes et al. (2006) [9] demonstrated that a low-carbohydrate diet lead to significantly more loss of LMM compared to a high unsaturated fat diet. The greater LMM loss during a low-carbohydrate diet may reflect an additional loss of water, a component of LMM [47]. Ketosis has been shown to cause water excretion [48], and in the current trial increased levels of βHB were associated with greater losses in LMM. Additionally, reduced insulin levels caused by ketosis, are known to inhibit proteolysis and may have therefore caused a reduction in LMM [49].

The majority of the literature reports a loss of 3%–5% in LMM during a low-carbohydrate diet [6,40,45] and a corresponding loss of bone mineral density [11], a factor associated with osteoporosis [46]. In contrast, LMM reduction in the current trial did not exceed 1.5% and we did not observe any significant changes in bone mineral density in the EX-LC group. Our results support previous studies which demonstrate that the addition of combined exercise training during low-carbohydrate diet is able to attenuate [50] or even completely eliminate the expected LMM loss [15]. This could be mostly attributed to the resistance exercise component of the exercise intervention and its effect on increasing muscle mass [51]. It is, however, difficult to quantify the need for muscle mass retention. A small decrease in total muscle mass may not necessarily be considered negative as obese individuals often have a larger total LMM than leaner counterparts [52]. Hence, loss of body weight will also reduce the load on the musculoskeletal system. With regard to muscle functionality and metabolic role, the quality of muscles may be considered more important than size.

### 5.3. Cardiometabolic Indices

In the present trial, both conditions resulted in marked improvements in total cholesterol, triglycerides, fasting glucose, CRP and blood pressure, indicating that the prescribed combined exercise intervention elicited beneficial reductions in cardiometabolic risk factors. Previous studies that have explored the effect of combined exercise in cardiometabolic indices have also demonstrated similar reductions in healthy older people and patients with metabolic syndrome [53,54]. Exercise-induced cardiometabolic adaptations such as increases in mitochondrial biogenesis [55] and vascular function [56] may attenuate insulin resistance and thus reduce cardiometabolic risk. Exercise-induced increases in mitochondrial biogenesis have been shown to have beneficial effects in lipidaemic profile, glucose uptake and anti-inflammatory pathways [57], which may explain the observations of the current trial.

In the current trial, participants in the EX-LC group demonstrated a greater reduction in inflammation documented by plasma concentrations of CRP, a marker associated with cardiovascular disease risk [58]. Although our results are consistent with the majority of previous studies involving obese individuals [59], there is little understanding or consensus regarding the role of carbohydrate restriction on inflammation. It has previously been suggested that βHB activates hydroxy-carboxylic acid receptor 2, a receptor responsible for lowering inflammation [60]. Given that in the current trial higher levels of βHB where associated with greater reductions in CRP, the above mechanism may explain our results. Further research would be required to better understand the role of carbohydrate restriction in modifying inflammation in overweight and obese individuals.

### 5.4. Limitations and Issues of Concern

We have to acknowledge that the current trial has several limitations. Firstly, based on the design of the current trial, it was not feasible to blind participants and provider, which is common in trials of such nature [9,10]. Additionally, recent evidence demonstrates that pre-prepared meals result in greater weight and fat loss than a standard self-selected diet [61]. However, due to feasibility reasons, this study was only able to provide the pre-prepared meals to the experimental condition (EX-LC). Both study groups demonstrated significant reductions in body fat. However, a greater body fat reduction was observed in those assigned to pre-prepared meals. This finding could be due to the reduced total energy intake induced by the portion controlled pre-prepared meals provided to the experimental group (EX-LC) compared to the standard self-selected diet suggested in the control (EX-CO) participants. We should further note that although we demonstrated that a low-carbohydrate diet is effective on reducing cardiometabolic factors in apparently healthy population, the appropriateness of reducing carbohydrate intake in clinical population such as patients with type 1 and 2 diabetes is still debated in the literature and requires further research.

Despite that this trial is demonstrating the beneficial effects of a short-term low-carbohydrate intervention combined with exercise training on cardiometabolic parameters, there are several hypothetical concerns of the long-term safety of low-carbohydrate diets that deserve mention. Incorporating more fat and protein in response to reduction of dietary carbohydrates has led to concerns about the effect of low-carbohydrate dieting on lipids—specifically, LDL cholesterol [62]. Recent systematic reviews of low-carbohydrate diets on lipids demonstrate a neutral to small increase in LDL, but a favourable triglyceride reduction, which was also the case in the current trial [7,8]. It is important to note that a subset of individuals with hyper LDL response to ketogenic diets has recently been identified [59]. Due to the varied and individualized response, baseline and periodic fasting lipid profile testing is recommended.

There have been several studies linking low-carbohydrate diets to increased mortality. Epidemiological studies and meta-analysis have shown an increased risk of mortality with carbohydrate intake < 40 g per day [63,64]. Contrariwise, a recent epidemiological study, involving over 135,000 participants globally, found a relationship between increased mortality and higher carbohydrate intake whilst, lower mortality was associated with higher fat intake [65]. Therefore, until long-term, randomized studies can be undertaken, the long-term effect of a low-carbohydrate diet on cardiometabolic parameters is unclear.

## 6. Conclusions

The current trial demonstrates that a short-term low-carbohydrate diet, combined with prescribed exercise training including aerobic and resistance exercise is able to elicit a significant improvement in the cardiometabolic profile of obese individuals. Combined exercise training may have also attenuated muscle mass loss, commonly observed with low-carbohydrate diets. Whereas the results of the current study are promising regarding the cardiometabolic benefit provided by a short-term low-carbohydrate intervention combined with exercise training, future studies should address the long-term effects of this dietary intervention on cardiometabolic parameters.

## Figures and Tables

**Table 1:** Baseline participant characteristics of the complete cohort and comparisons between the exercise program accompanied by standard dietary advice (EX-CO) or low-carbohydrate meals (EX-LC).

	ALL	EX-CO	EX-LC	*p* Value
	(n =64)	(n = 33)	(n = 31)	
**Participant Characteristics**				
Age (years)	35 ± 9	34 ± 8	35 ± 6	0.45
Height (m)	1.74 ± 0.3	1.75 ± 0.5	1.77 ± 1.1	0.16
Weight (kg)	87 ± 17	84 ± 16	86 ± 15	0.20
BMI (kg.m^−2^)	30.3 ± 3	30.8 ± 4	31.2 ± 3	0.17
Male Sex (%)	43	38	41	0.25

Data are presented as the mean ± SD; *p* < 0.05 indicates significant difference between conditions; BMI, body mass index.

**Table 2:** Changes in cardiorespiratory fitness, body composition and other cardiometabolic parameters following an 8 week prescribed exercise program accompanied by standard dietary advice (EX-CO) or low-carbohydrate meals (EX-LC).

	All		Time Effect	EX-CO		EX-LC		Condition Effect
	Baseline	Change	*p*	Baseline	Change	Baseline	Change	*p*
**Cardiorespiratory Fitness**								
*V*O_2peak_ (mL.kg^−1^.min^−1^)	28.1 ± 6.3	2.4 ± 3.7	0.001	28.9 ± 6.3	1.7 ± 2.9	27.7 ± 6	3.0 ± 4.5	0.002
**Blood Biomarkers**								
Total cholesterol (mmol/L)	4.9 ± 0.8	−0.3 ± 0.5	0.001	4.8 ± 0.8	0.0 ± 0.5	4.9 ± 1.0	−0.3 ± 0.6	0.830
HDL (mmol/L)	1.3 ± 0.4	0.0 ± 0.4	0.230	1.2 ± 0.3	0.0 ± 0.1	1.4 ± 0.4	0.0 ± 0.2	0.260
LDL (mmol/L)	2.9 ± 0.9	0.2 ± 0.5	0.092	2.9 ± 0.8	0.0 ± 0.6	2.9 ± 0.9	0.0 ± 0.5	0.622
Triglycerides (mmol/L)	1.4 ± 0.7	−0.3 ± 0.6	0.003	1.3 ± 0.5	0.0 ± 0.5	1.4 ± 0.9	−0.4 ± 0.7	0.045
Fasting glucose (mmol/L)	5.5 ± 0.4	−0.2 ± 0.3	0.005	5.4 ± 04	0.0 ± 0.4	5.6 ± 0.4	−0.2 ± 0.4	0.027
CRP (mmol/L)	1.7 ± 2.3	−0.6 ± 1.6	0.018	1.4 ± 2.2	−0.2 ± 1.3	2.0 ± 2.5	−0.9 ± 1.9	0.039
Adiponectin (ug/L)	23.0 ± 15	−0.8 ± 14	0.911	20.6 ± 14	1.3 ± 18	25.9 ± 15	−1.2 ± 9.5	0.417
**Blood Pressure**								
Brachial SBP (mmHg)	124 ± 10	−3.6 ± 6	0.004	121 ± 4	−3.3 ± 10	123 ± 7	−3.7 ± 7	0.800
Brachial DBP (mmHg)	82 ± 8	−3.5 ± 11	0.009	80 ± 7	−1.0 ±8	83 ± 9	−4.4 ± 7	0.092
**Body Composition**								
Body weight (Kg)	83.7 ± 18	−3.8 ± 21	0.001	84.6 ± 13	−1.8 ± 2.5	87.8 ± 16	−4.4 ± 4	0.007
Total body fat (%)	40.5 ± 6.6	−2.4 ± 5.7	0.001	40.0 ± 7.3	−1.3 ± 1.4	40.9 ± 6.0	−2.0 ± 2.0	0.090
Visceral adipose tissue (cm^3^)	114 ± 34	−15 ± 24	0.001	116 ± 39	−11 ± 15	116 ± 28	−13 ± 14	0.764
Lean muscle mass (cm^3^)	47,549 ± 10,366	−190 ± 2460	0.030	46,788 ± 9631	143 ± 976	48,106 ± 11,017	−854 ± 1670	0.020
Fat mass index (kg.m^−2^)	12.7 ± 5.4	−0.9 ± 0.9	0.001	13.1 ± 7.2	−0.6 ± 0.7	12.3 ± 2.8	−1.1 ± 0.9	0.013
Total bone mineral density (g/cm^2^)	1.106 ± 0.06	0.005 ± 0.00	0.025	1.116 ± 0.07	0.006 ± 0.01	1.102 ± 0.05	0.003 ± 0.00	0.659

Data are presented as the mean ±SD; time effect *p* < 0.05 indicates significant difference compared to baseline; condition effect *p* < 0.05 indicates significant difference between EX-CO and EX-LC conditions; HDL, high density lipoprotein; LDL, low density lipoprotein; CRP, C-reactive protein.

**Table 3:** Multiple linear regression analysis between ketosis and changes in cardiorespiratory fitness, body composition and other cardiometabolic parameters.

	b	95% CI	R	R^2^	*p*
**Cardiorespiratory Fitness**					
VO_2peak_ mL.kg^−1^.min^−1^	2.54	−1.78 to 6.87	0.252	0.064	0.026
**Blood Biomarkers**					
Total cholesterol (mmol/L)	−0.33	−0.73 to 0.44	0.068	0.015	0.618
HDL (mmol/L)	−1.33	−0.40 to 0.15	0.123	0.015	0.368
LDL (mmol/L)	−0.10	−0.47 to 0.67	0.048	0.002	0.724
Triglycerides (mmol/L)	−0.21	−0.86 to 0.50	0.190	0.036	0.161
Fasting glucose (mmol/L)	−0.28	−0.70 to 0.40	0.251	0.064	0.042
CRP (mmol/L)	−0.97	−0.56 to 1.58	0.245	0.060	0.041
Adiponectin (ug/L)	−3.20	−2.81 to 17.30	0.090	0.008	0.505
**Blood Pressure**					
Brachial SBP (mmHg)	−0.94	−6.96 to 5.07	0.040	0.002	0.755
Brachial DBP (mmHg)	−1.03	−6.40 to 4.33	0.050	0.002	0.701
**Body Composition**					
Total body fat (%)	−1.52	−0.35 to −2.68	0.317	0.101	0.011
Visceral adipose tissue (cm^3^)	−23,779	−3023 to −44,535	0.286	0.082	0.025
Lean muscle mass (cm^3^)	−7081	−250 to −13,913	0.261	0.068	0.042
Fat mass index (kg.m^−2^)	−0.90	−0.35 to −1.46	0.384	0.148	0.002

*p* < 0.05 indicates significant effect of ketosis; b, regression coefficient (data are presented in the same units as the measurement itself); CI, confidence interval; HDL, high density lipoprotein; LDL, low density lipoprotein; CRP, C-reactive protein.
